# Influence of the duration of brain death protocol on losses of potential donors by cardiac arrest

**DOI:** 10.1186/cc14696

**Published:** 2015-09-28

**Authors:** Miriam Cristine V Machado, Artur Montemezzo, Fernanda Cani, Gabriel Torres, Glauco A Westphal, Joel de Andrade, Leandro S Botelho, Tiago A Slaviero, Tiago C Carnin

**Affiliations:** 1 Central de Notificação, Captação e Distribuição de Órgãos do Estado de Santa Catarina (CNCDO/SC), Florianópolis, SC, Brazil

## Introduction

Organ transplantation is, in many cases, the only therapeutic option for patients with terminal organ dysfunction. Organ donation from deceased donors in brain death (BD) is the main source of organs for transplants. Goal-oriented protocols are associated with reduction of cardiac arrests in potential donors. However, there is not much information about chronological goals and the influence of the length of donor management on the loss of donors by cardiac arrest. The severe inflammatory reaction that occurs from the BD installation tends to amplify over time and could contribute to cardiovascular deterioration and occurrence of cardiac arrest.

## Objective

To evaluate the influence of the duration of brain death protocol (DBDP) on losses of potential donors by cardiac arrest.

## Methods

Retrospective analysis of potential donors notified to the Transplantation Center of the State of Santa Catarina in the period from May 2012 to April 2013. We determined the odds ratio of losses by cardiac arrest according to DBDP to identify the chronological threshold at which the probability of losses due to cardiac arrest increases. *p *values <0.05 were considered significant.

## Results

A total of 384 potential donors were analyzed and 272 were effective donors. There was a greater chance of cardiac arrests after the 30-hour threshold (OR 1.67, 95 % CI: 0.38-0.93) and the lowest risk of stops was identified in the range of 12-30 hours (OR 0.32, 95 % CI: 0.19-0.52). Multivariate analysis identified the following variables associated with cardiac arrest: potential donor management outside the ICU (*p *= 0.001), adherence to management checklist, DBDP up to 30 hours (*p *= 0.003) and DBDP between 12 and 30 hours (*p *= 0.004). Protocols lasting more than 30 hours had a higher percentage of cardiac arrests (35.4 % vs. 24.6 %, *p *= 0.022), and longer time intervals to perform the second clinical test (14:51 ± 13:02 vs. 9:46 ± 4:20, and 35:37 ± 29:43 vs. 11:36 ± 7:55, *p *<0.001).

## Conclusion

The long duration between the first clinical test for diagnosis of BD and the harvesting of organs may be an important risk factor for the occurrence of cardiac arrest in deceased organ donors.

